# Plasticity, elasticity, and adhesion energy of plant cell walls: nanometrology of lignin loss using atomic force microscopy

**DOI:** 10.1038/s41598-017-00234-4

**Published:** 2017-03-10

**Authors:** R. H. Farahi, A. M. Charrier, A. Tolbert, A. L. Lereu, A. Ragauskas, B. H. Davison, A. Passian

**Affiliations:** 1 Quantum Information Science, Computational Sciences and Engineering Division, Oak Ridge National Laboratory, Oak Ridge, TN 37830 USA; 2 BioEnergy Science Center (BESC), Biosciences Division, Oak Ridge National Laboratory, Oak Ridge, TN 37830 USA; 3 Department of Chemical and Biomolecular Engineering, University of Tennessee, Knoxville, TN 37996 USA; 4 Aix Marseille Univ, CNRS, CINaM, Marseille, France; 5 School of Chemistry and Biochemistry, Georgia Institute of Technology, Atlanta, Georgia 30332 USA; 6 Department of Physics, University of Tennessee, Knoxville, TN 37996 USA

## Abstract

The complex organic polymer, lignin, abundant in plants, prevents the efficient extraction of sugars from the cell walls that is required for large scale biofuel production. Because lignin removal is crucial in overcoming this challenge, the question of how the nanoscale properties of the plant cell ultrastructure correlate with delignification processes is important. Here, we report how distinct molecular domains can be identified and how physical quantities of adhesion energy, elasticity, and plasticity undergo changes, and whether such quantitative observations can be used to characterize delignification. By chemically processing biomass, and employing nanometrology, the various stages of lignin removal are shown to be distinguished through the observed morphochemical and nanomechanical variations. Such spatially resolved correlations between chemistry and nanomechanics during deconstruction not only provide a better understanding of the cell wall architecture but also is vital for devising optimum chemical treatments.

## Introduction

Being largely inert, nonreactive, and implicated in the structural integrity of plants, lignin is both sought-after^1^ and unwanted^2, 3^. Lignin removal is a high priority requirement for achieving efficient production of lignocellulosic biofuel^4^. Recalcitrance, the phenomenon associated with the reluctance of sugar complexes or polysaccharides, specifically cellulose and hemicellulose, to break free from their lignin network during biological deconstruction is a molecular engineering mastered by nature^2, 3^. The question of whether nanoscale alteration in the populations of the different molecular species, such as loss of lignin, would result in corresponding variations in the mechanical properties^5^ of the sample is particularly interesting in the investigation of chemical strategies^6^ for overcoming recalcitrance. The strength of a plant material describes a measure of its capacity to withstand forces acting on it without undergoing plastic deformation^7, 8^, i.e., irreversible changes of its shape. While polymers contained in lignocellulosic biomass lack long-range order, it is possible that the naturally woven molecules in the lignin, cellulose and hemicellulose may exhibit plasticity. However, acquiring the chemical signatures of compounds nondestructively at high spectral and spatial resolutions is challenging. Nanometrology^9–17^ and emerging optomechanical techniques^18^ can offer novel measurement modalities capable of achieving the needed morphochemical characterization. Consequently, to what extent mechanical properties can be used for achieving biomaterial characterization has been largely unexplored, in particular as a material characteristic to differentiate enzymatic degradation, acid treatment, and other chemical processing steps^19^. To achieve this, the mechanical properties measured must be able to bring about a required level of specificity of the varied chemical compositions. Quantitative nanomechanical characterization of native and *in situ* biomass can help in gaining a more predictive understanding of the complex biological processes relevant to energy and environmental initiatives. Relative to bulk measurements, analyzing such variations of pretreated biomass at the scale of decomposing bacteria and their enzymes will help understand their environment and guide improvements in pretreatment and bioprocessing. Here, we show how delignifying biomass leave distinctive morphochemical and nanomechanical traits. The significance of the presented work is that the invoked nanometrological tracking of the ultrastructures of the biomass, as it moves through the chemical processing stages, provides remarkable details of the cell wall architecture not previously visualized, reveals the extent of the lignin removal, and quantitatively provides variations in plasticity, elasticity, and adhesion energy.

## Results and Discussion

### Chemical treatments of delignification

**Figure 1:**
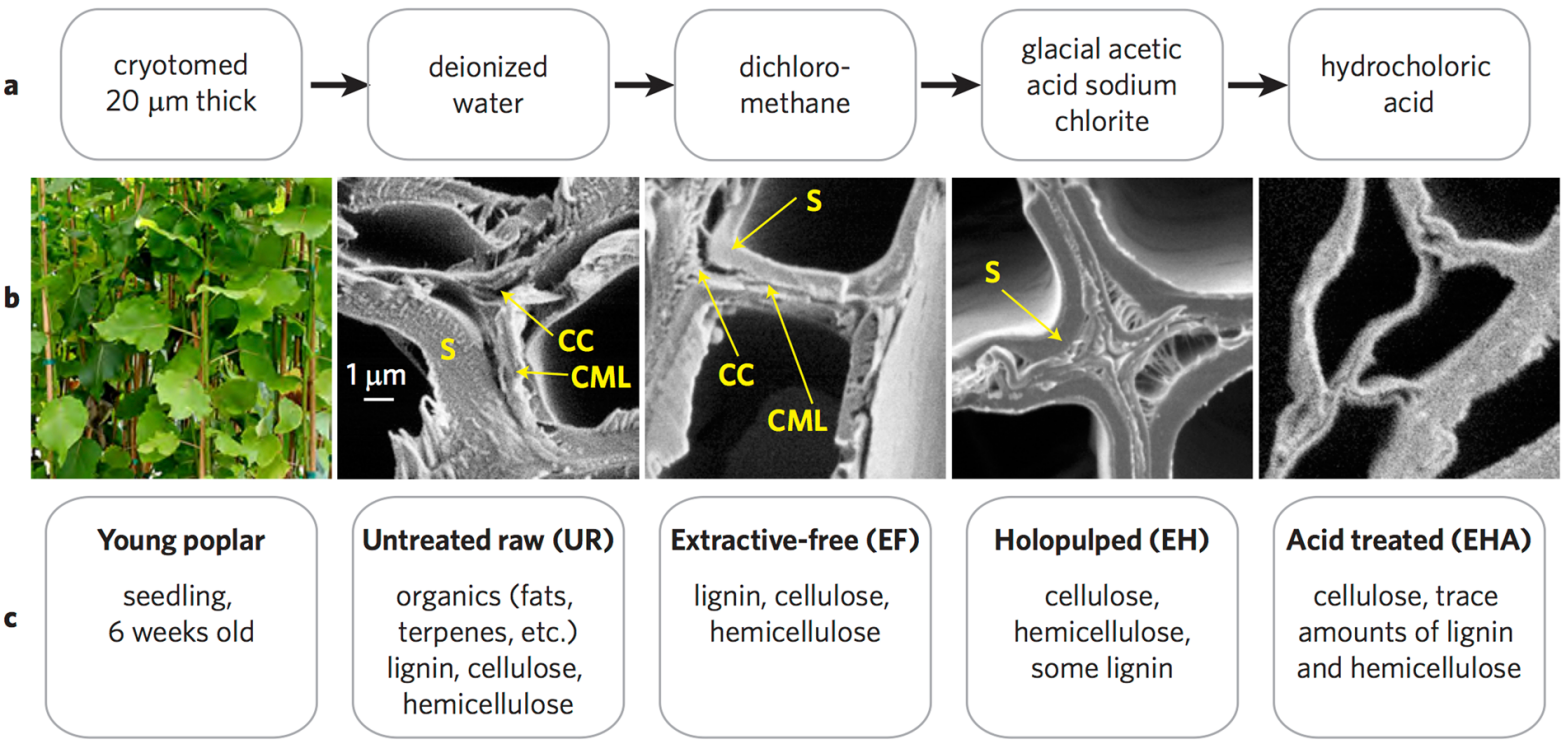
The sequence of chemical treatments for exploring the effect of lignin loss. (**a**) The sequence commences with untreated raw (UR) samples. Extractive-free (EF) samples are prepared by refluxing UR samples with dichloromethane followed by treatment with glacial acetic acid and sodium chlorite. The acid treatment (EHA), following the extractive-free holopulped (EH) samples completes the sequence. (**b**) The investigated plant species and the scanning electron microscope images (5000X magnification) of the samples at each stage of the chemical processing. (**c**) Annotation of the lignocellulosic content corresponding to UR, EF, EH, and EHA. See the supplementary materials.

In order to execute lignin loss, we carried out a sequence including holopulping and acid treatment to remove extractives, lignin, and hemicellulose from 20 *μ*m thick poplar cross-sections, resulting in untreated raw poplar (UR), extractive-free (EF) poplar, extractive-free holopulped (EH) poplar, and extractive-free holopulped acid-treated (EHA) poplar, shown in Fig. 1. Following the sequence displayed in Fig. 1, prior to chemical reactions, UR samples were produced from a young poplar (*Populus deltoides*) stem. The removal of extractives allows for the cellulose and lignin to be detected with less barrier on the plant cell walls by ToF-SIMS and other characterization methods. Extractive-free holopulped samples are produced from sodium chlorite treatment, which essentially bleach and breaks up the lignin, leaving behind the cellulose and hemicellulose. The solubilization of lignin can occur in various ways, including degradation of side chains, demethylation, and oxidation of quinones. The final step of acid treatment leaves behind alpha-cellulose (EHA sample).

### Preliminary compositional measurements and analysis

**Figure 2:**
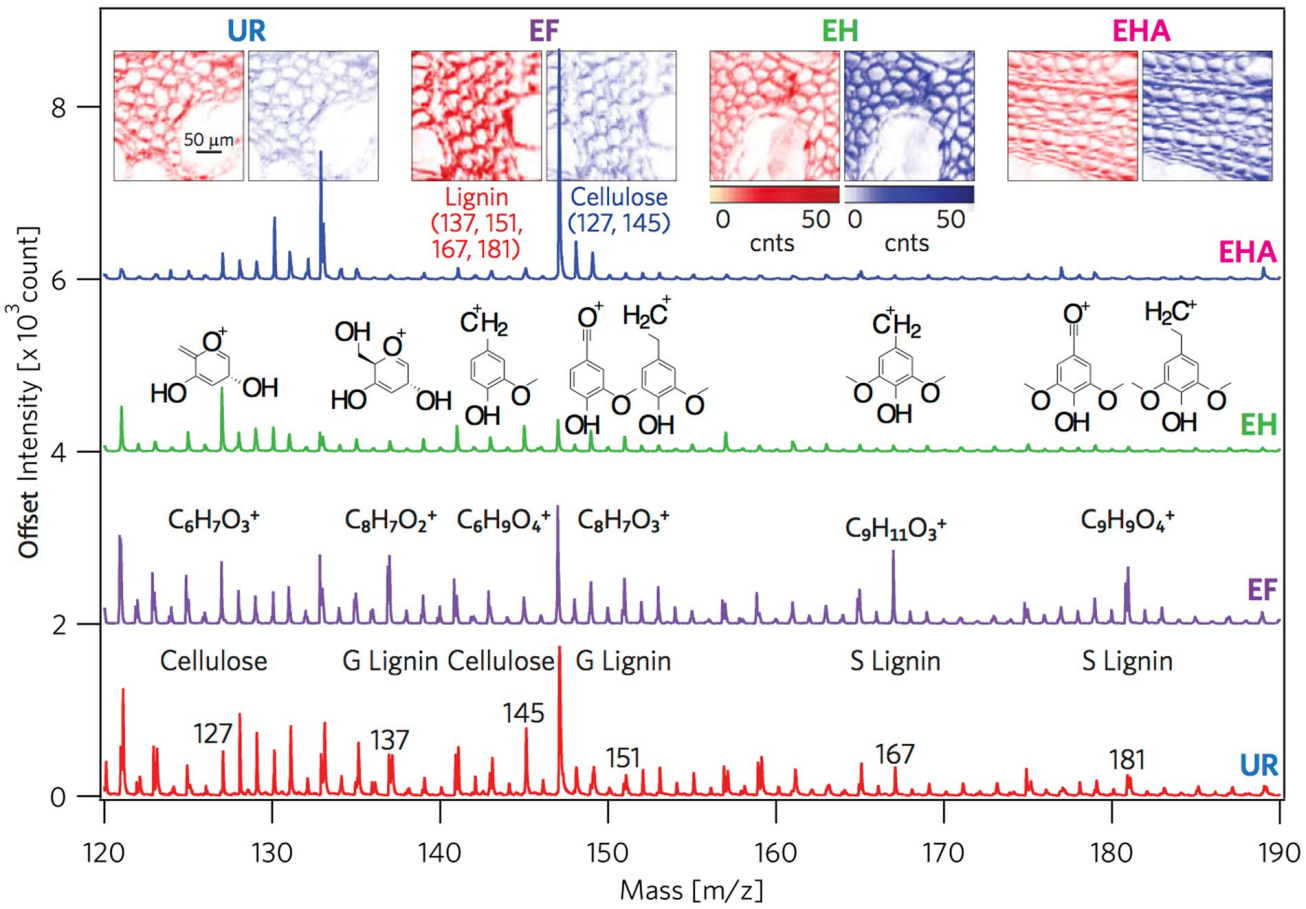
Tracing delignification by identifying cellulose, G lignin, and S lignin ion currents. The cellulose (*m*/*z* = 127 and 145) and lignin (*m*/*z* = 137, 151,167, 181) intensities of secondary ion mass spectra (offset separated) are displayed with their molecular structures. The image insets show the spatial mapping of the sum of the lignin and cellulose components for each processing step. See the supplementary materials for details and the *m*/*z* assignments.

**Figure 3:**
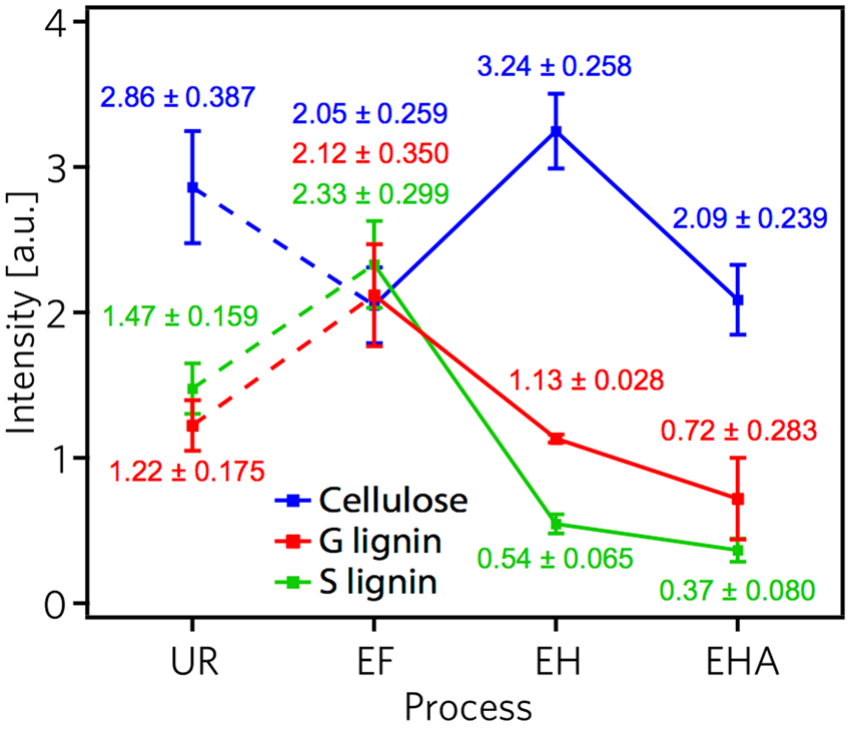
Delignification compositional trend by averaged scaled spectral values. Process driven content variations corresponding to UR, EF, holopulp EH, and alpha-cellulose EHA samples for the cellulose, guaiacyl (G) lignin, and syringyl (S) lignin ion fragmentations. The dashed lines indicate the UR sample content is not prepared to compare, whereas the solid lines compare the contents following extractive removal.

The surface composition of the samples is expected to undergo changes during each step of the treatment, described in Fig. 1b. Employing ToF-SIMS, mass spectra (Fig. 2) were analyzed and relative ion intensities (Fig. 3) were determined. The fragmentation ions of the major components of biomass are cellulose, guaiacyl (G) lignin, and syringyl (S) lignin^20, 21^. Cellulose units are C_6_H_7_ ${{\rm{O}}}_{3}^{+}$ and C_6_H_9_ ${{\rm{O}}}_{4}^{+}$ with mass-to-charge ratios *m*/*z* = 127 and 145, respectively. G lignin fragments to *m*/*z* = 137 (C_8_H_9_ ${{\rm{O}}}_{2}^{+}$ C_8_H_9_ ${{\rm{O}}}_{2}^{+}$) and 151 (C_8_H_7_ ${{\rm{O}}}_{3}^{+}$), while S lignin yields *m*/*z* = 167 (C_9_H_11_ ${{\rm{O}}}_{3}^{+}$) and 181 (C_9_H_9_ ${{\rm{O}}}_{4}^{+}$) (see the supplementary materials). In the inset of (Fig. 2) cellulose and lignin ion fragmentations peaks were mapped to show spatial locations of the components. Figure 3 summarizes the average normalized ion intensities, where samples in the UR state are noted not to have been chemically prepared for comparison. However, ion intensities can be reasonably compared once samples are prepared in the EF state. Analyzing the results, the scaled ion intensities for G and S lignin from EF to EH are determined to have decreased by 62.3% and 76.7%, respectively. A reduction of lignin is also observed after the EH sample underwent acid treatment, where G lignin dropped by 35.1% and S lignin decreased by 32.6% from EH to EHA. Even though the holopulp and acid treatments appear not to have completely removed lignin, significant reduction of the lignin was observed on the surface of the sample. The holopulping is intended to oxidatively solubilize the lignin, leaving behind the cellulose and hemicellulose. As a result of the holopulping, a 58.5% increase in cellulose ion intensities was observed compared to the extractives-free sample. Interestingly, reduction in cellulose was also found after the acid treatment from the ToF-SIMS analysis.

### Investigating material domains

**Figure 4:**
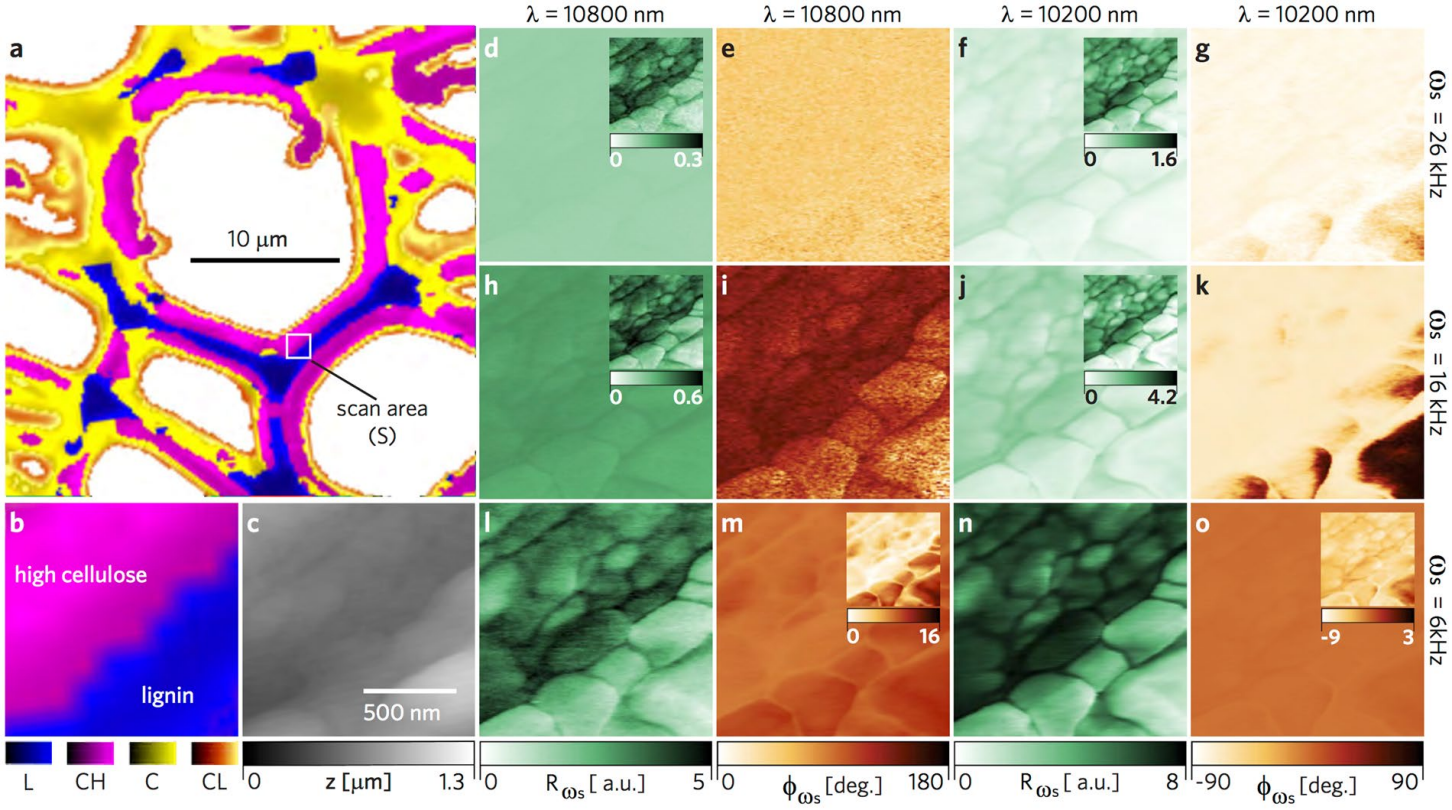
Photoacoustic force microscopy of cellulose and lignin. Employing PAS-AFM at a low absorption channel at *λ* = 10800 nm and a high absorption channel at *λ* = 10200 nm, the sample (EF) actuation rate was controlled from an amplitude modulated quantum cascade laser yielding sample oscillation frequencies *ω*_*s*_ = 26 KHz, 16 KHz, and 6 KHz. (**a**) Raman image independently identifying lignin and cellulosic content. The square scan area marked as S locates the L–CH transition region over which images (**b**–**o**) were acquired. (**b**) Corresponding Raman image over region S. (**c**) Topography of S with contact mode AFM (height z). The amplitude ${{\rm{R}}}_{{\omega }_{s}}$ (**d**,**h**,**l**) and the corresponding phase ${{\rm{\Phi }}}_{{\omega }_{s}}$ (**f**,**j**,**n**) responses at low absorption. The amplitude ${{\rm{R}}}_{{\omega }_{s}}$ (**d**,**h**,**l**) and the corresponding phase ${{\rm{\Phi }}}_{{\omega }_{s}}$ (**f**,**j**,**n**) responses at high absorption. Each image group in the right four columns (images (**d**–**o**)) maintains the same scale. The insets display the substructures at the highest contrast, although the signals are weaker. Lower sample actuation frequency yields a stronger signal in accordance with the sample relaxation; distinction between the material domains is amplified at high absorption.

In order to independently identify a heterogeneous region of lignocellulosic biopolymers, we first carried out confocal Raman spectroscopy of EF at a coarse resolution of 320 nm. In Fig. 4a, the material domains from clusterization are mapped according to Raman bands^22, 23^ and their signal strength, designated as L (lignin, blue), CH (high cellulose signal, pink), C (cellulose signal, yellow), and CL (low cellulose signal, orange). See supplementary materials for the average spectra corresponding to each cluster. The molecular regions also correspond to the cell wall structure: 1) the cell corners (CC) and compound middle lamella (CML) in blue having a strong lignin signal, and 2) the layers of the secondary wall (S) in pink having a distinct cellulose/hemicellulose signal. The yellow and orange regions are also interpreted to be of cellulosic content. Specifically, cellulose and hemicellulose together exhibit dominant bands near 330, 380, 1095, and 2900 cm^−1^ bands for EF, accounted by the overlapping vibrational modes of CCC, CO, and CCO ring deformation (329 cm^−1^), heavy atom bending (331 cm^−1^), heavy atom stretching (380 cm^−1^), CC and CO stretching (1095 cm^−1^), CH and CH_2_ stretching (2889 cm^−1^) and CH stretch (2917 cm^−1^). The lignin heteropolymers exhibit overlapping peaks of syringol-based and guaiacol-based monomers around the aromatic lignin band at 1600 cm^−1^, dominated by the aromatic C-C vibration. (See the supplementary materials). Further characterization of the scan area (S), encompassing L and CH domains, shown in Fig. 4b for the Raman signal, compared to the image in Fig. 4c acquired with atomic force microscopy (AFM), demonstrates that lignocellulosic domains cannot be unambiguously distinguished through topography alone. Capitalizing on the infrared (IR) absorption signature of bulk lignin and cellulosic materials, we employed photoacoustic spectroscopy atomic force microscopy (PAS-AFM) method to provide localized distinction of the cell wall. Applying an amplitude modulated IR beam from a quantum cascade laser (QCL) source (range 10.1 *μ*m–10.93 *μ*m) at the probing site, we reveal compositional differences from the local sample response, as well as subsurface structural variation (Fig. 4d–o). Selecting the highest (*λ* = 10.2 *μ*m) due to the C-O stretching and lowest (*λ* = 10.8 *μ*m) absorption and setting three modulating frequencies (*ω*_*s*_ = 6 kHz, 16 kHz, and 26 KHz) of the QCL source, the amplitude (${{\rm{R}}}_{{\omega }_{s}}$) and phase (${\varphi }_{{\omega }_{s}}$) responses are shown for S in Fig. 4d–o. The amplitude images in Fig. 4d,h,l,f,j,n and phase images in Fig. 4e,i,m,g,k,o are plotted at the same contrast scale, respectively, for a more transparent comparison of the *λ* and *ω*_*s*_ setting, whereas the insets show the same images plotted at relative scale. More contrast of the lignocellulosic domain is revealed with the higher absorption wavelength and lower frequency modulation along with additional detail of the cell wall structure and the cellulose-lignin globular aggregates in the secondary wall^24, 25^, with the amplitude response at *λ* = 10200 nm (980.39 cm^−1^) in Fig. 4n having the strongest signal. Even with little absorption at 10800 nm (Fig. 4d,e,h,i,l,m), the minute changes by photoacoustic stimuli are detected by the probe, providing structural features not found in the AFM image.

### Domain boundaries at high resolution

**Figure 5:**
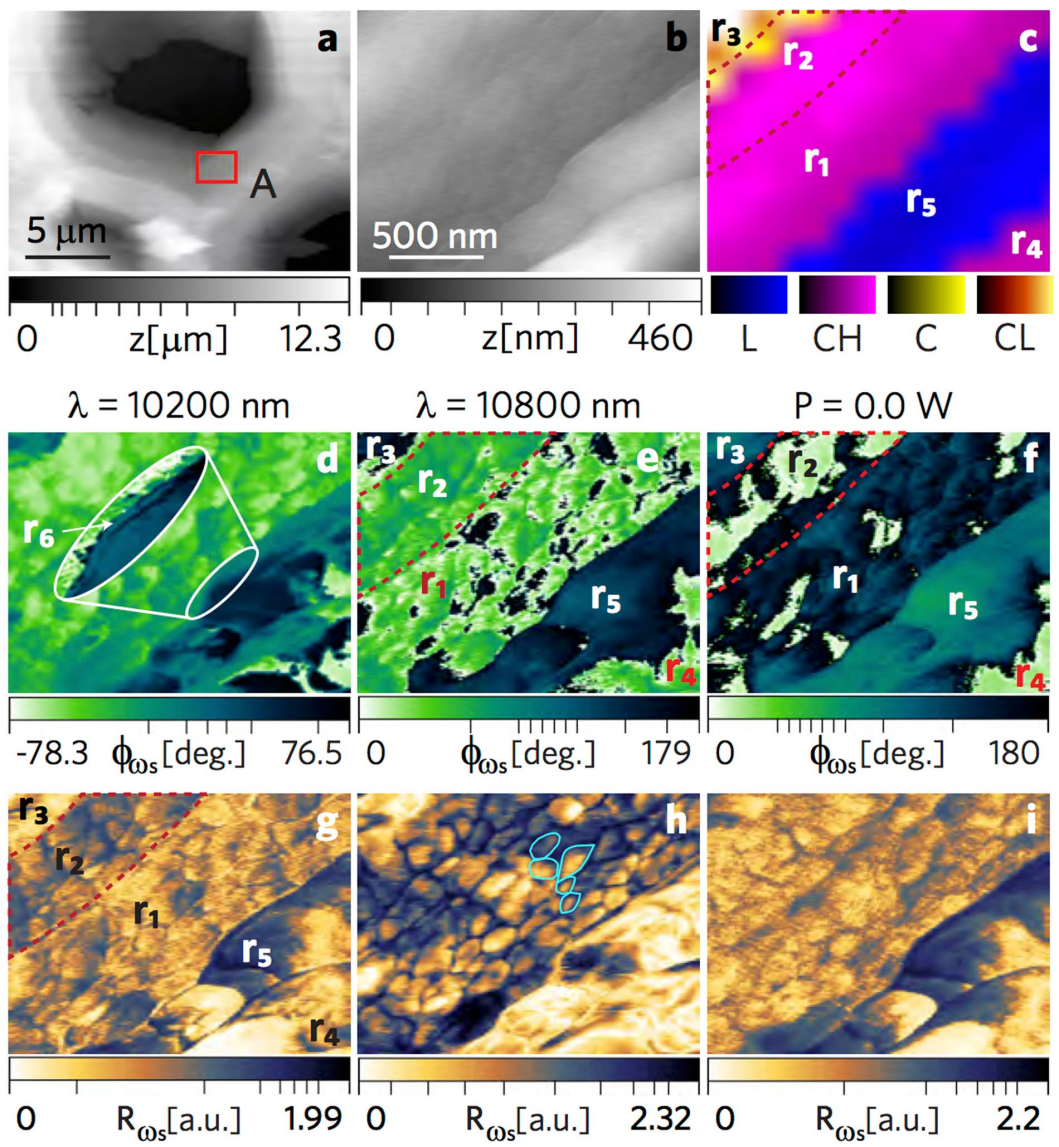
Resolving cell wall ultrastructure and chemistry. HPFM image acquisition via frequency mixing involving photoacoustic sample (EF) actuation and cantilever multifrequency oscillations. The photoacoustic sample actuation was achieved using an amplitude modulated quantum cascade laser tuned to a low absorption channel at *λ* = 10800 nm and a high absorption channel at *λ* = 10200 nm. (**a**) Contextual AFM image indicating the examination area, A. (**b**) Topography of A with contact mode AFM (height z). (**c**) Raman image over A with marked regions, r_1_ through r_5_, of the cell sturcture. (**d**,**e**,**f**) Phase ${\varphi }_{{\omega }_{s}}$ images at a laser wavelength of *λ* = 10200 nm and *λ* = 10800 nm at P = 1.3 mW, and P = 0 mW (no photonic actuation), with region r_6_ identified in (**d**) (see the supplementary materials). (**g**,**h**,**i**) Corresponding amplitude images to the above phase images, with 100 to 200 nm wide cellulose agglomerates outlined in (**h**).

Although the performed PAS-AFM provides localized nanospectral information of the sample, the distinction of the lignocellulosic domains of the cell walls can be significantly improved by invoking the hybrid-photonic nanomechanical force microscopy (HPFM), revealing the primary wall, secondary walls, and microfibril aggregates that have not been seen before using this technique^14, 18^. Here, we chose a basic mode encompassing probe forcings with frequencies ${\omega }_{{p}_{1}}$ and ${\omega }_{{p}_{2}}$ and a sample (photoacoustic) forcing *ω*_*s*_. Thus, a new mode of probe oscillation is generated at the difference frequency $({\omega }_{-}=|{\omega }_{{p}_{1}}-{\omega }_{{p}_{2}}|)$ that is further mixed with the frequency of sample oscillation to create high resolution chemical and subsurface maps. The cell wall is topographically mapped by AFM in Fig. 5a. An area of further examination, A, is imaged by AFM in Fig. 5b and Raman spectroscopy in Fig. 5c. The HPFM images of A are shown in Fig. 5d–i (plotted in nonlinear scale) when applying mechanical excitations of *ω*_*p*,1_ = 3.316 MHz and *ω*_*p*,2_ = 3.300 MHz (synthesized mode *ω*_−_ = 16 kHz), and photonic stimulation of *λ* = 10200 nm (high absorption), *λ* = 10800 nm (low absorption), and laser off (no absorption) at modulation frequency at the synthesized mode *ω*_−_ → *ω*_*s*_ = 16 kHz. In HPFM, the optomechanical response of the sample yields a signal with useful amplitude and phase content. The amplitude (${R}_{{\omega }_{-}}$) and phase (${\varphi }_{{\omega }_{-}}$) signals are observed to capture different features of the cell wall. Whereas the amplitude signals appear sensitive to the response of the near-surface structures (globules), the phase signal appears to be sensitive to the cell wall substructure (secondary, CML regions, etc) throughout the thickness of the sample. Therefore, we argue that phase images appear more sensitive to the cell wall architecture^26^, whereas the amplitude images appear to better display the variations in the cellulosic domain of the secondary wall.

The phase maps in Fig. 5d–f are strikingly sensitive to the lignocellulosic domains compared with the AFM (Fig. 5a,b) and Raman (Fig. 5c) assessments, identifying the CML in region r_5_, the S_1_ outer layer in region r_1_ (580 nm wide) and r_4_, the middle layer S_2_ in region in r_2_ (440 nm wide), and the inner layer S_3_ in region r_3_. The region r_2_ is based on the phase image in Fig. 5f, corroborated by Fig. 5e,g. The right side boundary can be identified in Fig. 5d–g, while the left side boundary is determined from a combination of Fig. 5e,g and to a lesser extent by Fig. 5i. We believe the ability of the signal to identify r_1_, r_2_, and r_3_ originate in the microfibril angle (MFA) of secondary wall layers having various angular configurations, as described by Barnett and Bonham^27^, contributing to distinct mechanical properties detected by the HPFM's sensitivity to subsurface heterogeneity. As the differences in MFA have a profound effect of the stiffness of wood^27^, and HPFM is sensitive with respect to the angle of measurement of the microfibrils (similarly for AFM nanoindentation^28^), the HPFM can be an important characterization tool for many biological materials where heterogeneous, anisotropic elastic properties are present. Interestingly, when *ω*_−_ = 26 kHz, we discovered another domain, labeled *r*_6_, as shown in the inset of Fig. 5d. Based on the location and the scale, we identify r_6_ in the phase image to be the primary cell wall, measured at approximately 10 nm thick. (See the supplementary materials). The maps in Fig. 5g–i are more revealing in the fine details of 100 to 200 nm sized globular aggregates of cellulose fibrils and lignin (Fig. 5h) of the secondary layer where a matrix of cellulose chains and lignin create the aggregates^24, 25^. As can be seen in these maps, there is significant heterogeneity within each domain, and the amplitude graph in Fig. 5h allows identifying clearly the presence of agglomerates. Interestingly, a different type of heterogeneity appears in Fig. 5e, which may be linked to the different information that the phase and the amplitude contain. The lower absorbing photonic excitation (*λ* = 10800 nm) in Fig. 5e,h was sufficient to draw out the mechanical and chemical heterogeneities of the cell wall, whereas the high absorbing mode (*λ* = 10200 nm) appears to saturate the HPFM signals. With no photonic contribution in Fig. 5f,i, the images are dominated by the nanomechanical component (essentially a single mode of mode synthesizing atomic force microscopy (MSAFM) ref. 10), but provide detail of the cell wall structure and substructure without chemical distinction. Chemical composition can only be acquired with photonic actuation (Fig. 5d,e), where the secondary layers (r_1_, r_2_, r_3_, r_4_) are collectively distinct from the CML (r_5_).

### Wideband optomechanical analysis

**Figure 6:**
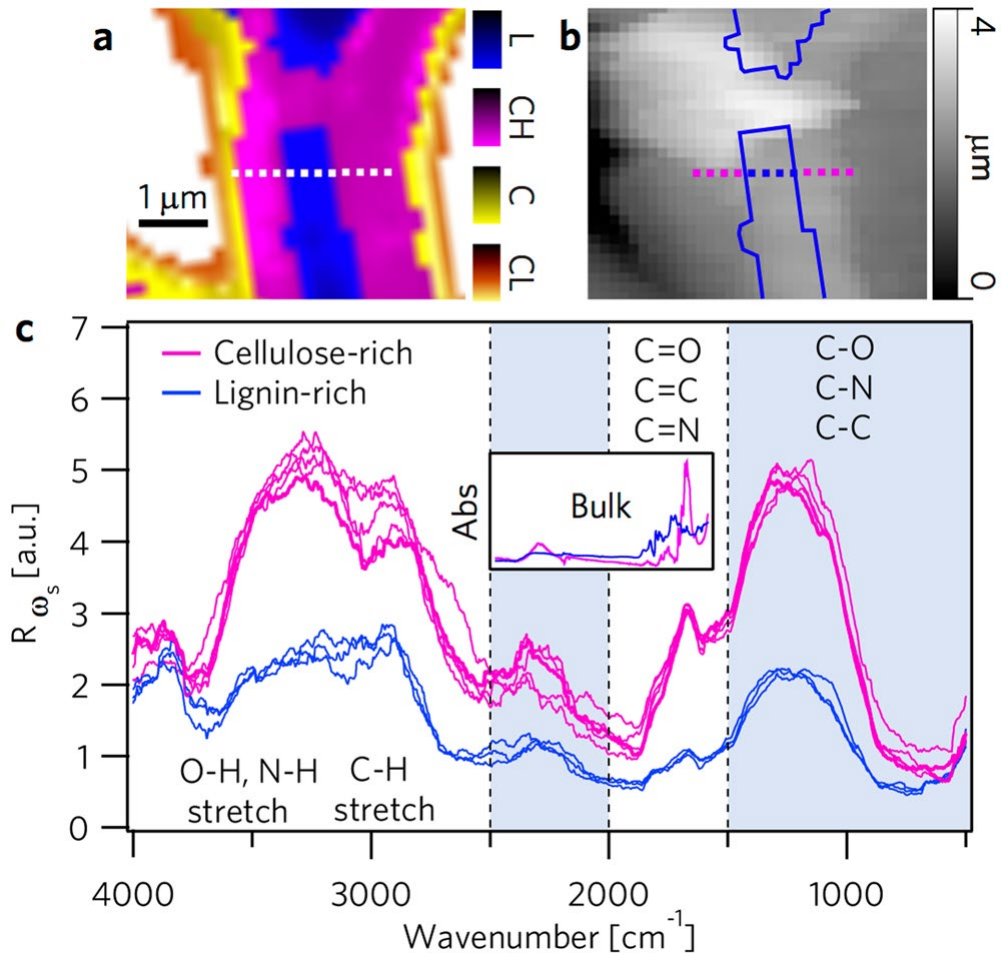
Hyperspectral interogration across cell wall by wideband infrared nanospectroscopy. (**a**) Raman image of EF prepared by cluster analysis. The square markers of 200 nm pitch indicate where nano-spectra were acquired across a cell wall cross section. (**b**) A corresponding topographic image acquired by AFM, where lignin areas are outlined and the square markers are color-coded based on the material composition. (**c**) The wideband nanospectra acquired through PAS-AFM produces a characteristic difference in signal strength between cellulose and lignin (color assignment based on probe location). For comparison, the infrared (bulk) absorption spectra of cellulose Avicel and lignin acquired by FTIR, plotted at the same range of 4000 to 500 cm^−1^, are shown in the inset.

In order to demonstrate the spectral origin of the PAS-AFM (Fig. 4) and the HPFM images (Fig. 5), we demonstrate how a given data point on the images represents only a narrow band from a rich spectrum available at that location. Lignocellulosic composition can be monitored by the relative changes at 4000–2500 cm^−1^ (single bond stretch), 2000–1500 cm^−1^ (double bond), and 1500–500 cm^−1^ (fingerprint) regions of the absorption spectra^29, 30^. Using PAS-AFM, where photonic excitation by a wideband thermal source is delivered to the sample at the probe measurement point, IR absorption spectra were acquired across known lignocellulosic regions of EF, shown in Fig. 6. In Fig. 6a Raman spectroscopy reveals the spatial location of the lignocellulosic biopolymers in the cell wall, which is outlined over the corresponding AFM image in Fig. 6b. Discrete locations of the nanospectra acquisition are indicated by the small squares (200 nm step size) across a cell wall, where at each step the spectral response of the sample due to the wideband excitation are color-coded as pink or blue based on the Raman cluster region. In Fig. 6c the nano-spectra, match remarkably well with the independent Raman analysis, where the group of spectra of higher magnitude occurs in the cellulosic region and the group of low magnitude spectra falls into the lignin-rich region. (See a a similar response for QCL in the supplementary materials). These spectrally distinct nanoscopic regions, indicative of varied lignin and cellulose content, may be expected to correspondingly exhibit mechanically distinct nanoscopic regions. More importantly, what role will the loss of lignin play in modifying these distinct regions has remained elusive.

### Mechanical signatures of lignin reduction

**Figure 7:**
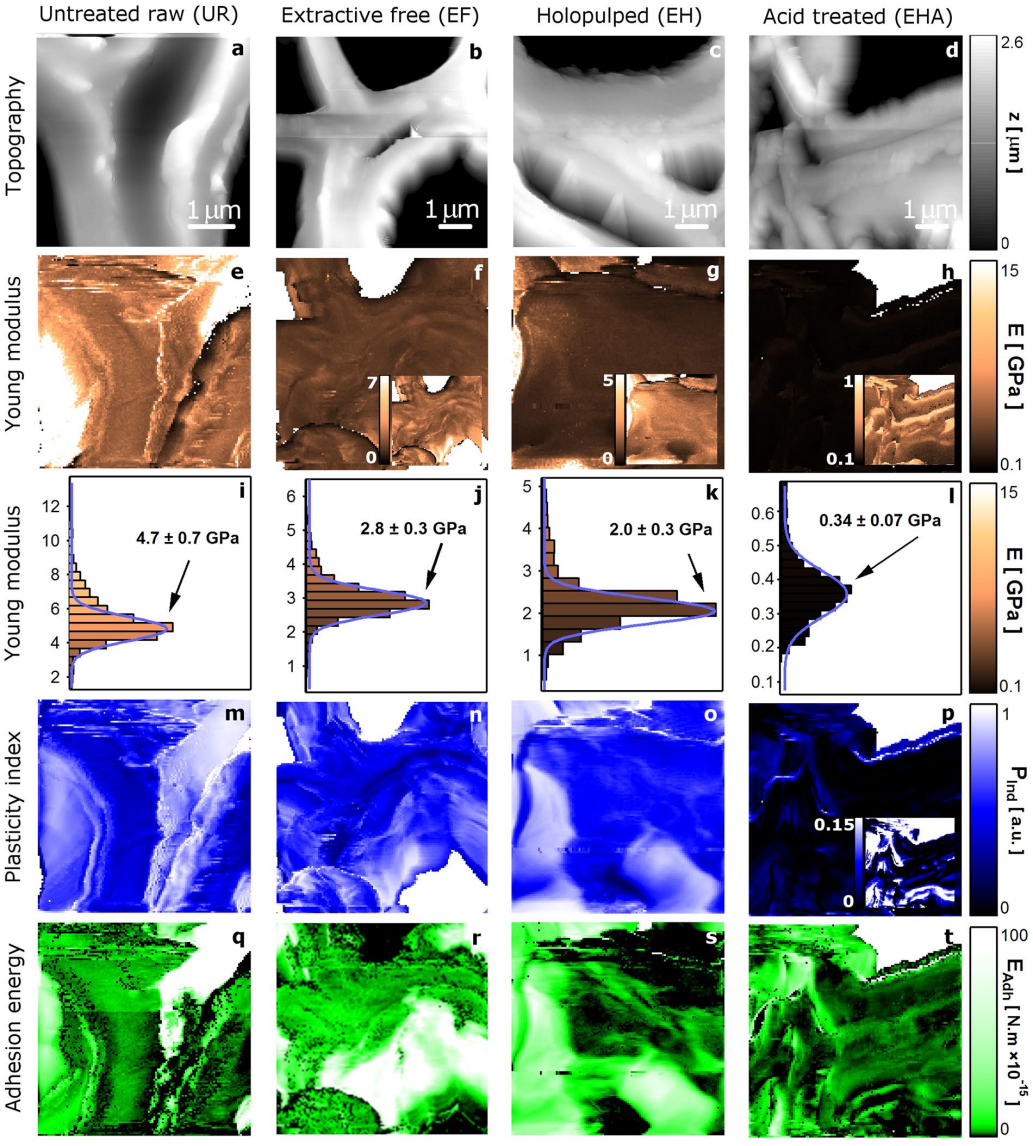
Tracing the physical traits of the delignification sequence. (**a**–**d**) Measurement of the topographic effects on the treated cell walls by AFM. (**e**–**h**) Mapping of the elasticity variations due to lignin removal. The contrast presents computed Young moduli assuming Sneddon model. The vastly reduced Young moduli is notable in the EHA case. (**i**–**l**) Distribution of the computed Young moduli *E* indicating the loss of elasticity caused by the drop in lignin content. (**m**–**p**) Observation of plasticity index ${\rm{\Psi }}$ where plastic-like behavior emerges due to lignin removal. (**q**–**t**) Mapping the localized surface adhesion energy as lignin is lost.

To visualize nanoscale details of the structural and material differences of the cell wall as it progresses through the delignification process, we carry out AFM of the UR, EF, EH and EHA samples (Fig. 1), and obtain both topographic information and a volume of force curves as the probe (indentor) interacts with the surface, as shown in Fig. 7a–d. We note that nanoindentation measurements on cell walls are highly dependent on the direction of forces applied by the indentor. We examined cross sectioned samples where the forces are parallel to the direction of the plane of the cell wall and the axial growth of the stem. Nanoindention forces applied normal to the plane of primary walls have been used to study polysaccharide-lignin content and distribution in whole cells^31, 32^. Analyzing the measured forces, we compile plasticity, elasticity, and adhesion energy of the cell wall, as shown in Fig. 7e–t. The elasticity (Fig. 7i–l) is observed to be systematically reduced throughout the chemical process with Young moduli (*E*) of 4.6 ± 2.3 GPa (UR), 2.8 ± 0.1 GPa (EF), 1.9 ± 0.1 GPa (EH), and 0.35 ± 0.02 GPa (EHA). Noting that the plasticity index of ${\rm{\Psi }}=1$ yields a fully plastic material, while ${\rm{\Psi }}=0$ corresponds to a perfectly elastic response, we observe that ${\rm{\Psi }}$ (Fig. 7m–p) drops significantly with HCl treatment in EHA, which may be expected since hemicellulose, having smaller branched chains (lower degree of polymerization) than cellulose, is hydrolyzed by HCl at the 1,4- and 1,3-linkages thus solubilizing it. Furthermore, we note that beta- and gamma-cellulose are more easily dissolved than alpha-cellulose, leaving primarily alpha-cellulose in EHA^33–35^ where inter- and intra-molecular hydrogen bonding between cellulose chains forms crystalline structure. Assuming the crystallinity of alpha-cellulose is essentially preserved during the degradation process of lignin removal in EHA (similiar to bulk studies^36, 37^), the observed reduction in plasticity and elasticity suggests the alpha-cellulose microfibrils have lost its biomechanical structure and natural axial orientation, due to the missing network of lignin monomers. Because nanoindentation is particularly sensitive to cellulose microfibril orientation^28^, the localized fibril disorder or disorientation caused by the delignification process may be quantified through *E* mapping. It is important to acquire *E* over a sufficiently large domain so that the distribution can be quantified (the histograms in Fig. 7i–l) and comparisons can be made^38^. Plant cell walls being aniostropic due to the orientation of the cellulose microfibrils^39^, (the force and deformation vectors of the local nanoindentation of the cell wall being positioned in the axial direction normal to the cell wall cross section), we expect that measured values can vary depending on specimen orientation. Similarly, the mapping of the adhesion energy (Fig. 7q–t) shows a reduction along the successive sample treatments with values ranging from 0 to 10^−13^ N · m. Prior to lignin loss (UR and EF), it is reasonable to assume that a higher degree of surface contact is available for the probe-sample interaction due to the higher density of the surface molecules, where these molecules are held together by the van der Walls forces between lignin molecules and cellulose fibrils^40^. As the lignin breaks free from the cellulose fibrils due to the holopulping and acid treatment, the extent and spatial distribution of the dissolution of the lignin can be quantified by the adhesion energy measurements. Thus, having identified distinct molecular regions with HPFM, the relationship between elastic properties and sample composition, can be ascertained by nanomechanical measurements.

**Figure 8:**
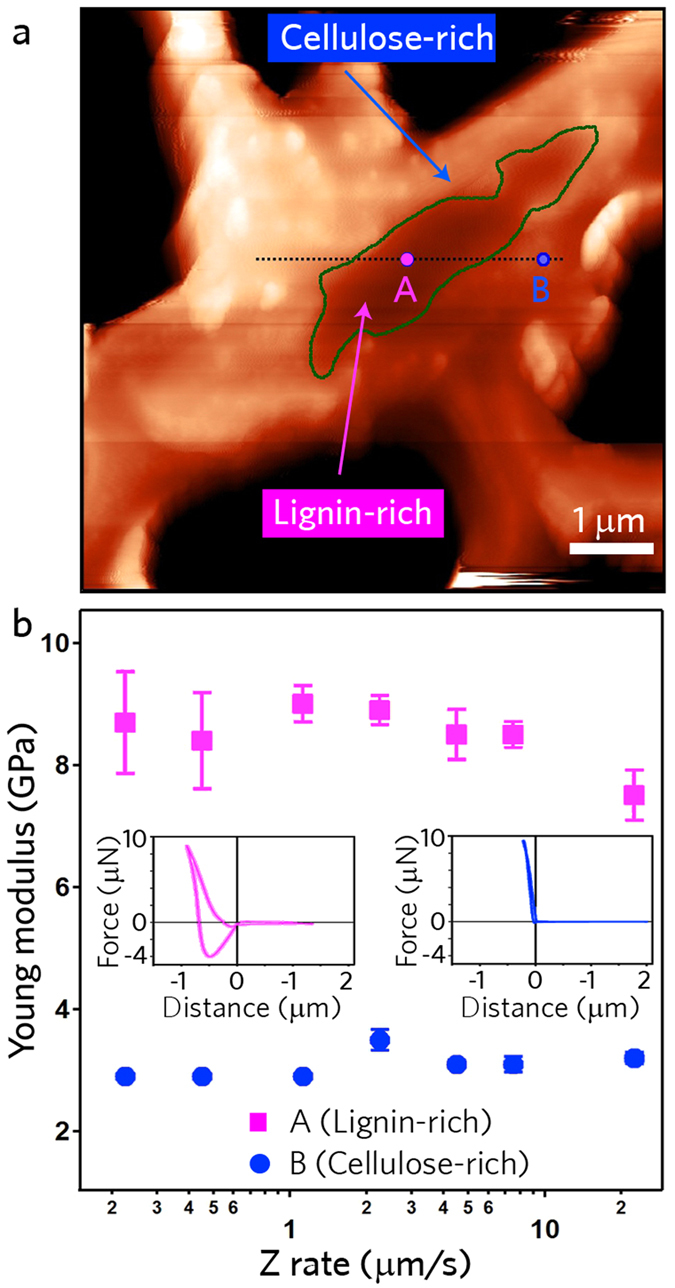
Material differentiation of UR solely through nanomechanical properties. (**a**) Topographic image acquired by AFM with perimeter of lignin-rich region of the cell corner lamella marked with interrogations across the material domains, specifically points A and B. (**b**) The mean Young modulus of 25 measurements at different positions, with error bars representing the overall distribution (standard deviation). Lignin-rich domains were measured to be *E* = ~8 GPa and for cellulose-rich *E* = ~3 GPa. The insets are a representative force-distance measurement for the two material domains.

In order to show the underlying tip-sample interaction that is the basis for the high resolution nanomechanical images in Fig. 7, we present discrete approach curves across distinct material domains in Fig. 8. Delimited by the green line on the Fig. 8a, distinct mechanical behavior is observed for UR. In contrast to the force curve of the cellulose-rich region (right inset of Fig. 8b), displaying highly elastic behavior, the force curve of the lignin-rich region (left inset of Fig. 8b) exhibits energy dissipation and adhesion occurring during the indentation process, allowing, with post-process analysis, these material domains to be identifed through adhesion energy. The origin of the observed dynamic dissipation was explored by altering the indentation rate in the range 0.2 to 20 *μ*m/s and at each rate acquiring an average of 25 measurements per point in Fig. 8. While important differences are observed in the Young moduli, with a value of ~3 GPa for the cellulose and ~8 Gpa for the lignin, no appreciable dependence on the loading/unloading rate was measured, ruling out material viscosity. See supplementary materials for additional force curves. These results support our previous conclusion that the removal of lignin from the samples along with the different chemical treatment leads to a decrease of the Young moduli of the cell wall. These observations also strengthen the conclusion that nanomechanical properties by themselves can be of potential to differentiate the various materials.

## Concluding Remarks

Following the loss of lignin through the deconstruction of lignocellulosic biomass, we find that the cell walls’ nanomechanical properties undergo a quantifiable reduction in plasticity, adhesion energy, and elasticity. The unprecedented molecular recognition of the cell wall chemical composition over spatially resolved morphological features using HPFM, without special sample preparation or labeling, was essential in identifying the secondary wall components, primary wall, compound middle lamella, globular aggregates, and lignin/cellulose domains. Interestingly, the reduction in plasticity may seem counterintuitive considering that lignin adds rigidity to the cell wall. Nanomechanical mappings may quantify the extent of dissolution of the lignin, where the breakdown of the carbohydrate complex may be argued to occur concurrently with adhesion energy reduction. Spatially resolving the consequent changes of the cell wall structure can lead to establishing a correlation between morphochemistry and nanomechanics. This is feasible since lignocelluosic domains exhibit sufficient mechanical diversity, allowing the chemical processes of the delignification to be tracked through quantitative measurement of the Young moduli, plasticity index, adhesion force, and adhesion energy.

Despite the observations of the nanoscale mechanical variations, the question of whether it is possible to distinguish which are due to the chemical treatment and which are the result specifically of lignin depletion warrants further study. Such distinction would likely require devising challenging control experiments to isolate the effect of chemical treatment without the loss of lignin and/or the removal of the lignin without chemical treatment. The outcome of such control experiments would shed light on to the role of *in situ* lignin: 1) a chemical composition and structure, and 2) a functional capacity within the plant. Compositional analysis of the lignin phenolic group types can be obtained, for example from Raman spectroscopy, however, the physical/biological condition of the sample may still be unknown. Changes to the functional aspect of lignin, such as resulting in the alteration of the cell wall structural integrity, may be observed with nanomechanical spectroscopy via AFM. Emerging nanometrology, such as the HPFM, provides a combination of compositional and physical information but requires proper signal analysis.

The origin of the observed reduction in elasticity may receive contributions from multiple effects. It is conceivable that the reduction may be linked to the extent by which the cellulose fibril is altered or disoriented in the cell wall as lignin is lost, as well as to the outright removal of lignin and hemicellulose constituents. In addition to the potential role of loss of biomechanical structure, the origin of the loss in plasticity may be argued to be due to the absence of flexible polymers. A considerable loss in plasticity seems to occur only with acid treatment, implying it could be strongly coupled to hemicellulose hydrolysis rather than lignin removal.

Similarly, the dramatic loss of plasticity with acid treatment may be viewed as a result of two related events from hemicellulose hydrolysis. First, one may consider the biomechanical breakdown of the lignocellulosic structure occurring concurrently with the removal of these polymers. In this case, the lignin and hemicellulose polymers no longer contribute to a matrix that forms a normal cell wall, now thought to possess lower plasticity due to the remaining disoriented cellulose microfibrils. Second, we may consider simply a removal of the constituent polymers (lignin and hemicellulose) that are thought to contribute to the overall properties of the cell wall. While the plasticity properties of the individual constituents are largely unknown *in situ*, it is conceivable that the sheer absence of these two polymers, can lead to loss of plasticity. In this case, the loss of plasticity could be interpreted as primarily due to the loss of the hemicellulose, which is amorphous compared to the strong lignin and crystalline cellulose components.

## Methods

### Sample preparation for chemical treatment and cell wall characterization

Prior to chemical reactions, a young poplar (*Populus deltoides*) stem was frozen and sectioned with a cryotome using a disposable blade that was free of any lubricant. To maintain structural integrity of the cell walls, all samples were stored and dried between glass slides after any chemical treatment. Initially, the freshly cut cross-sections were washed with DI water and some were set aside as UR (untreated raw) samples. EF (extractive free) poplar samples were prepared by refluxing FP samples with dichloromethane (CH_2_CL_2_, an organic solvent) for 6–12 h to remove the extractives. Extractives are non-structural components in lignocellulosic biomass that can be removed by washing or refluxing with water or neutral organic solvents. The EF samples were treated with glacial acetic acid (C_3_COOH, a weak acid) and sodium chlorite (NaClO2) at 70 degrees for 2 h. They were then filtered and rinsed twice with DI water to produce the EH (extractive-free holopulped) poplar samples. The sodium chlorite is used to form ClO^−^, essentially bleach, which breaks up the lignin, leaving behind the cellulose and hemicellulose. The solubilization of lignin can occur in different ways, including degradation of side chains, demethylation, and oxidation of quinones. It is important to note that it does not completely solubilize the lignin. To obtain *α*-cellulose (EHA sample), holopulp was treated with 2.5 M hydrogen chloride (HCl) at 100 degrees for 4 h, which allowed the HCl to solubilize the hemicellulose at the 1,4- and 1,3- linkages. The residual solids were then filtered and rinsed with DI water, leaving cellulose-rich samples with traces of lignin and hemicellulose remaining.

### ToF-SIMS for surface elemental analysis through the chemical sequence

The Poplar samples were analyzed using a ToF.SIMS 5 (Muenster, Germany) and Measurement Explorer ionTOF software. The high mass resolution spectra were obtained by randomly rastering a ${{\rm{Bi}}}_{3}^{++}$ primary ion beam across a 500 *μ*m^2^ area at 200 scans and 128 × 128 pixels. The fragmentation ion peaks correlating to cellulose, G lignin, and S lignin were selected from the calibrated spectra and normalized in relation to the total ions detected (see the supplementary materials). The average normalized ion intensities were determined by observing 5 randomly selected locations on the sample (the EHA sample had 4 detection sites). The high spatial resolution images were formed by rastering the Bi_3_ primary ion beam across areas ranging from 20 *μ*m^2^ to 100 *μ*m^2^ with 200 scans at 256 × 256 pixels.

### Raman microscopy for spectral analysis through the chemical sequence

Confocal Raman images of the samples were acquired with a WITec Alpha500R Near Field Scanning Optical Microscope (NSOM) at an excitation wavelength of 532 nm. Using a 100X objective (NA 1.25) a source beam was delivered and formed a spot size of 330 nm that defines the system spatial resolution. At each raster point in the 100 × 100 point scan at a step size of 20 *μ*m, a 1600 point Raman spectrum was obtained at 3 cm^−1^ resolution using a 600 g/mm grating blazed at 500 nm. To avoid thermal breakdown of the chemical bonds caused by higher absorption at the source laser wavelength, the laser power was kept below 50 mW.

### Force-distance and nanoindentation metrology for physical analysis through the chemical sequence

To study the elastic and plastic properties of the samples, we realized quantitative force-volume mapping on samples cross sections using NTEGRA AFM system from NT-MDT. In all experiments AFM tips NSC15 from Mikromash were used with typical resonant frequency of 150 kHz, spring constant in the range 22–32 N/m and apex radius of 8 nm as verified by scanning electronic microscopy. For each tip, the spring constant was determined before and after the measurements using the thermal noise method after obtaining the deflection sensitivity of the cantilever by pressing the AFM tip against a hard reference silicon surface. Mapping of the Young modulus was extracted from discrete force curve measurements realized at each point of a 120 × 120 array by computing a model based on the Oliver and Pharr method using the Sneddon contact mechanics assuming a conical tip in contact with a flat surface^41^. In all cases the adhesion was found to be generally smaller than 10% of the applied loading force and was considered negligible in the model.

## Electronic supplementary material

Supplementary information
