# Supplementary material for: Plasticity, elasticity, and adhesion energy of plant cell walls: nanometrology of lignin loss using atomic force microscopy
Source: Sci Rep. 2017 Mar 10;7:152. doi: 10.1038/s41598-017-00234-4 (PMC5428038; doi:10.1038/s41598-017-00234-4)
Supplement: Supplementary file 1 — Supplementary information [file 41598_2017_234_MOESM1_ESM.pdf]

Supplementary Materials for Plasticity, elasticity, and adhesion energy of plant cell walls: nanometrology of lignin loss using atomic force microscopy

R. H. Farahi[1,2,3], A. Charrier[4], A. Tolbert[2,5], A. L. Lereu[1], A. Ragauskas[2,3], B. H. Davison[2,3], and A. Passian[1,2,3,6,*]

[1]Quantum Information Science, Computational Sciences and Engineering Division, Oak Ridge National Laboratory, Oak Ridge, TN 37830, USA
[2]BioEnergy Science Center (BESC), Biosciences Division, Oak Ridge National Laboratory, Oak Ridge, TN 37830, USA
[3]Department of Chemical and Biomolecular Engineering, University of Tennessee, Knoxville, TN 37996, USA
[4]Aix Marseille Université, CNRS, CINaM UMR 7325, 13288 Marseille, France
[5]School of Chemistry and Biochemistry, Georgia Institute of Technology, Atlanta, Georgia 30332, USA
[6]Department of Physics, University of Tennessee, Knoxville, TN 37996, USA
[*]passianan@ornl.gov

ABSTRACT

Supplementary materials.

ToF-SIMS

Time-of-flight secondary ion mass spectrometry (ToF-SIMS) detects lower molecular weights of monomers, unlike matrix-assisted laser desorption ionization (MALDI) and electrospray ionization (ESI) which mainly characterize macromolecules[1,2]. ToF-SIMS bombards the sample surface with primary ions resulting in secondary ions flying off, and detects the positive and negative ions. It is capable of detecting the surface of solid samples without any treatment. In addition, this imaging mass spectrometry can interpret different types of data, for instance, high mass resolution spectrum and high spatial resolution images. Semi-quantitative analysis is available using ToF-SIMS analysis software through determining the normalized ion counts/intensities for specific mass peaks of desired ion fragmentations in the spectra. Moreover, spatial mapping of specific ion fragmentation masses provides insight into relative high or low concentration areas of the selected chemical fragment. The ToF-SIMS is also capable of depth profiling the sample by producing a series of spectra and/or a three-dimensional image[3,4].

ToF-SIMS has been used to study a variety of samples, including proteins on biodevices[5], bone[6], cancer cells[7], and a multilayer drug capsule. This is possible due to the instrument's capability of detecting both organic and inorganic ions. Recently, the application of ToF-SIMS expanded to map the cellulose and lignin in a plant cell wall[3,8,9]. The key to successful analysis is in the sample preparation by making the sample section as flat and smooth as possible, which might require the sample, like rat brains, to be embedded in material prior to sectioning[10]. There are also a number of different ways to dry the samples, including freeze dry and freeze fracturing[10]. The freeze fracture samples are necessary when targeting highly mobile K^+ and Na^+; analyzing the samples at cryogenic temperatures minimizes their movement[10].

The untreated raw (UR), extractives-free (EF), holopulp (EH), and α-cellulose (EHA) air-dried cross-sections were analyzed using a ToF.SIMS 5 (Muenster, Germany) and Measurement Explorer ionTOF software. ToF-SIMS library of fragmentation ions of the major biomass components was used for the analysis. Image of EH at different resolutions are presented in Fig. S.1. High mass resolution data and spatial resolution images were obtained via ToF-SIMS analysis at 100 μm^2 $\times$ 100μm^2, 256 x 256 pixels, and 200 scans. The images were formed from the sum of cellulose and lignin ion peaks in the spectra. The fragmentations along with the chemical structures associated with each component (cellulose, G lignin, and S lignin) are presented in Table S.1.

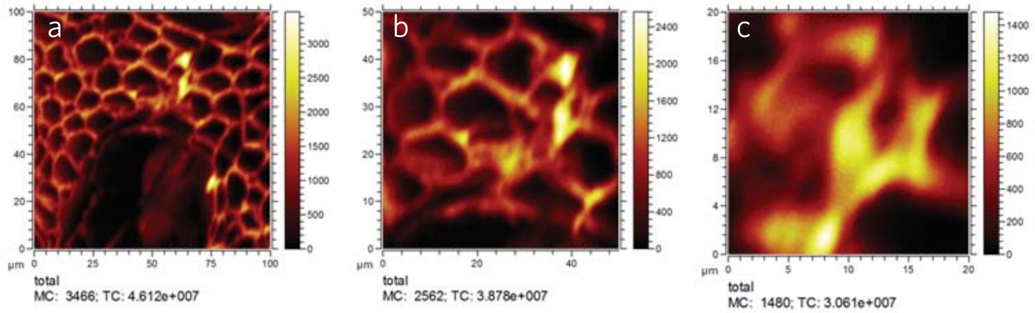

Figure S.1. ToF-SIMS total ion images of EH. Images at successive zooms. **a** 100 $\mu m^2 \times 100\mu m^2$. **b** 50 $\mu m^2 \times 50\mu m^2$. **c** 20 $\mu m^2 \times 20\mu m^2$.

Cellulose	*m/z* 127	$C_6H_7O_3{}^+$	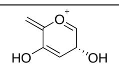
	m/z 145	$C_6H_9O_4{}^+$	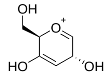
G Lignin	*m/z* 137	$C_8H_9O_2{}^+$	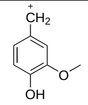
	m/z 151	$C_8H_7O_3{}^+$	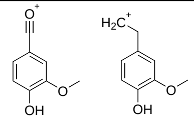
S Lignin	*m/z* 167	$C_8H_9O_2{}^+$	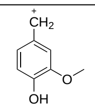
	m/z 181	$C_8H_7O_3{}^+$	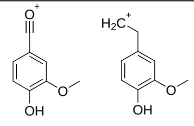

Table S.1. ToF-SIMS ion fragmentation for cellulose, guaiacyl (G) lignin, and syringyl (S) lignin.

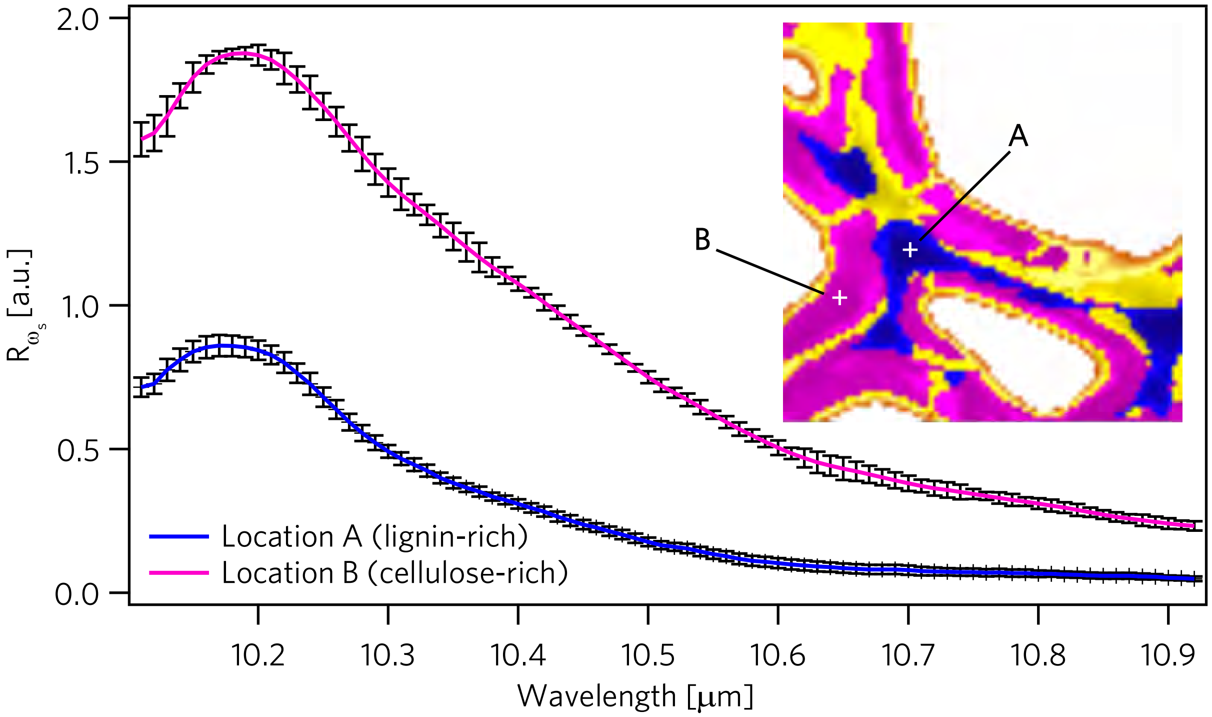

Figure S.2. Isolation of photothermal response. Plots of R_{ω_s} excited by QCL on EF over λ =10.10 to 10.93 μm and ω_s = 16 KHz. The probe is located at a lignin-rich location (A) and cellulose-rich location (B) of extractive-free Poplar. The mean and error bars were calculated from 20 measurements.

Photothermal signal

In order to study the underlying photothermal processes, we isolated the photothermal signal in Fig. S.2 at two locations on extractive-free Populus, shown in the Raman image inset: location A (lignin-rich) and location B (cellulose-rich). The probe remained stationary for 20 measurements at each location (A and B) across the range of the QCL (10 μm). The mean (solid lines) and standard deviation (error bars) show a fairly stable and repeatable HPFM signal, where the overall HPFM difference between a cellulose-rich region and a lignin-rich region are sufficiently larger than the error, making chemical distinction possible. Using this information, we are able to readily identify cellulose/lignin regions in biomass samples. The results for cellulose and lignin are consistent with those obtained using wideband sources.

Scanning electron microscopy images

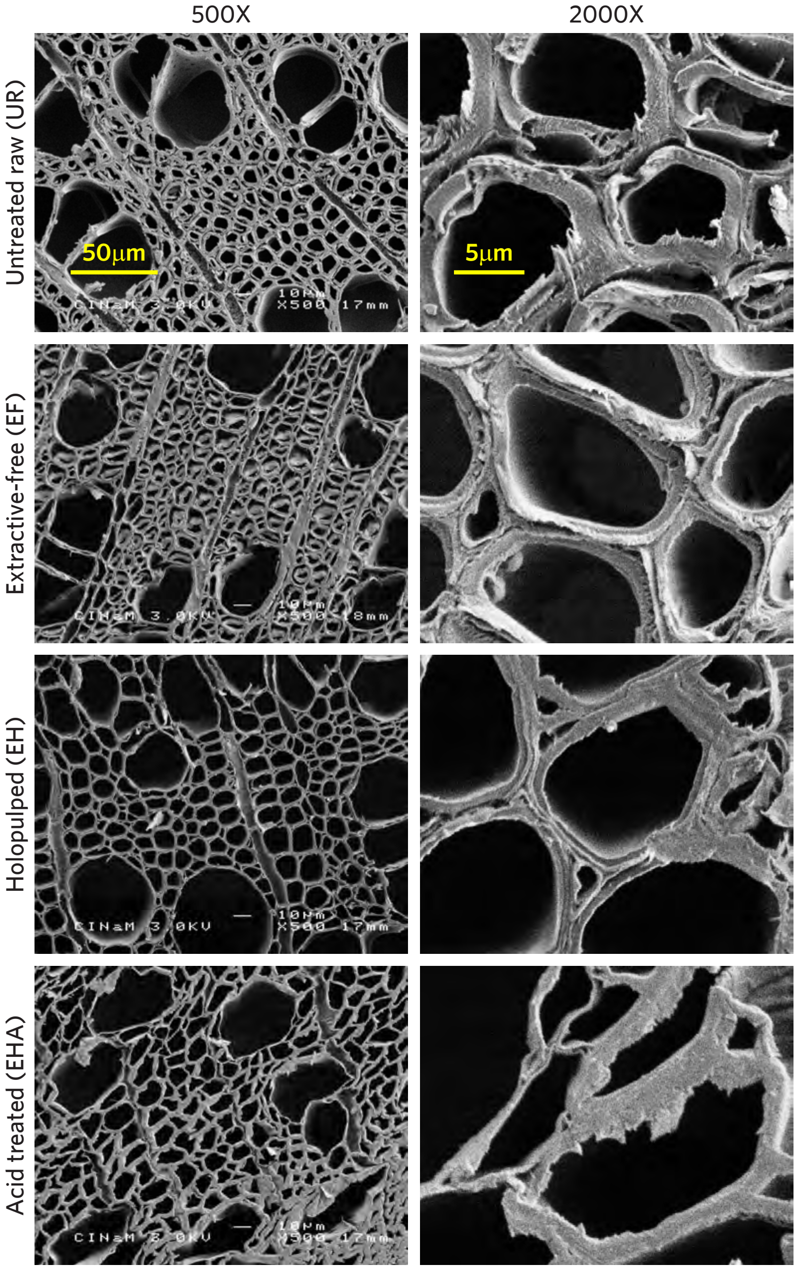

Figure S.3. **SEM images of *Populus* samples** Top row: SEM images taken at 500X magnification. Bottom row: SEM images at 2000X magnification.

Confocal Raman analysis

Our measured peaks for *Populus* are shown in Table S.2. The raman spectra of the lignin-rich and cellulose-rich locations for each sample are shown in Fig S.4. Cluster analysis is used to separate the cell wall regions in the Raman images; the corresponding spectra for the clusters are shown in Fig S.5. The possible indication of the classic fermi resonance doublet at 1278 and 1385 cm^{-1} were found.

Major assignment	Vibrational mode	Processed samples UR	EF	EH	EHA
		Raman shift (cm^{-1})			
Cellulose	Heavy atom str	384	384	384	384
	Heavy atom str	435	435	435	–
	CC, CO str	972	972	972	972
	CC, CO str	1049	–	–	–
	CC, CO str	1099	1099	1099	1099
	CC, CO str	1125	1120	1120	1128
	CC, CO str and HCC, HCO bend	**1154**	**1146**	–	–
	HCC, HCO bend	1278	1278	1278	–
	HCC, HCO bend	**1337**	**1337**	**1337**	**1337**
	CC Str	**1383**	**1383**	**1383**	**1382**
	HCH, HOC bend	1463	1454	1464	1471
	CH, CH_2 str	2897	2895	2895	2897
	CH str	2940	2940	2940	–
Cellulose, Triglycerides, Steryl esters, Fatty acids, Resin acids, Sterols	CH str	2897	2895	2895	2897
Lignin	C=C vib	**1600**	**1600**	1600	–
Triglycerides, fatty acids	CC str	908	–	905	910
Unsaturated fatty acids, resin acids, sterols	C=C str	1657	1664	1669	–
Triglycerides, steryl esters	C=C str	–	–	1736	–
		Unassigned peaks 384, 435, 523, 3370			

Table S.2. Detected Raman peaks. The major band positions and chemical assignments for cellulose, lignin, and extractives, and mapping to the peaks measured for the *Populus deltoides* samples studied, where bold type indicates strong peaks.

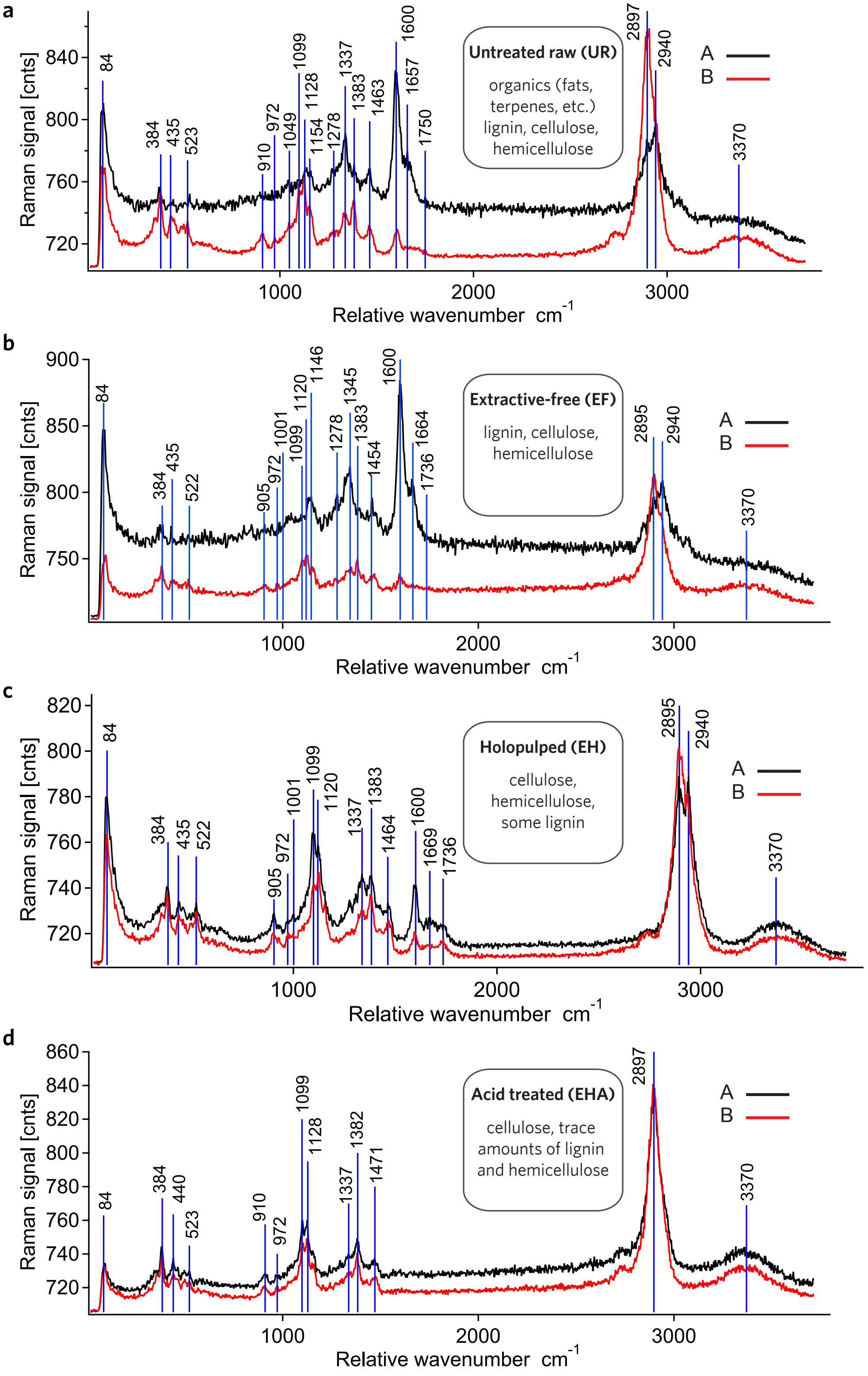

Figure S.4. Raman spectra of lignin-rich and cellulose-rich locations. On the processed *Populus* samples, Raman spectra at cellulose-rich (black) and lignin-rich (red) locations are shown: **a** untreated raw *Populus* (UR), **b** extractive-free *Populus* (EF), **c** extractive-free holopulped (EH), **c** and extractive-free holopulped acid-treated (EHA). The measured peaks are summarized in table S.2.

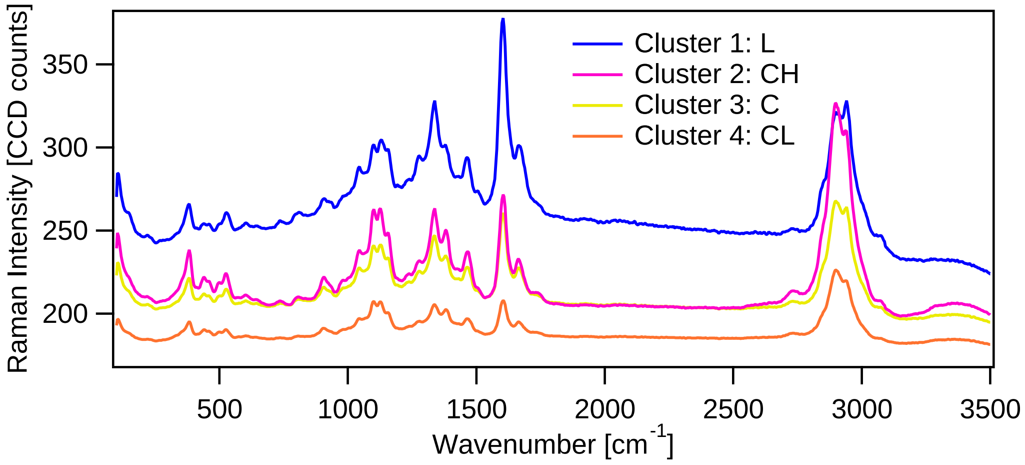

Figure S.5. Corresponding average spectra of the cluster analysis. The constructed Raman images differentiate the cell wall structure based on the Raman bands. Cluster 1 is designated as lignin-rich with characteristic C=C vib peak at 1600 cm^{-1}. Clusters 2-4 are designated as cellulose-rich with characteristic peaks summarized in table S.2.

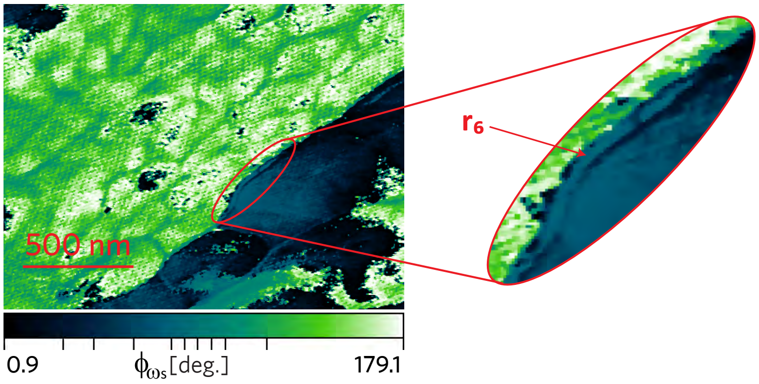

Figure S.6. Visualization of cellular ultrastructure with HPFM. Phase ϕ_{ω_s} image when applying mechanical excitations of $\omega_{p,1}$ = 3.326 MHz and $\omega_{p,2}$ = 3.300 MHz (synthesized mode ω_{-} = 26 kHz), and photonic stimulation of λ =10200 nm. The marked region is enlarged to show the r_6 domain, which we identify to be the primary cell wall based on the location and dimensions.

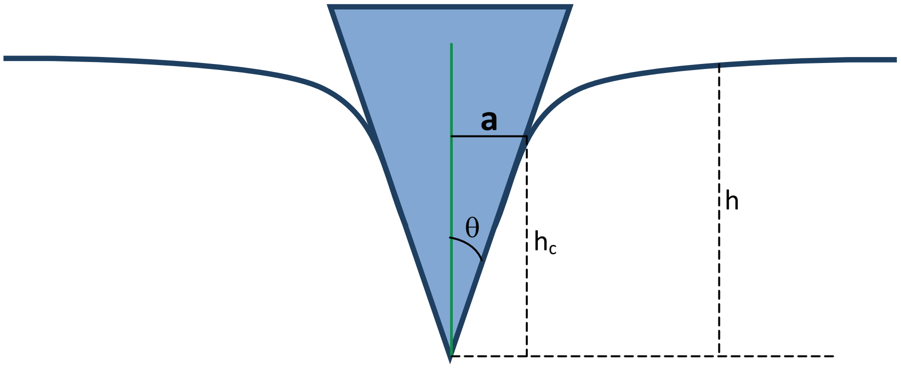

Figure S.7. Nanoindentation with AFM. A conical tip of angle θ indents into the surface a distance h with a contact height h_c. The radius of the tip at h_c is a.

Force measurements

A technique for measuring mechanical properties of solid materials is nanoindentation[11,12]. The technique entails pushing an indenter (AFM probe tip) with a known shape into a material surface, and measuring the loading force and the penetration depth as the indenter is aproached and retracted. Using numerical methods, various information (plasticity, elasticity, adhesion) can be extracted from quantitative force-volume measurements acquired during a localized indentation event. In our case, we employed an in-house program based on the Oliver and Pharr method using the Sneddon model of a conical tip in contact with a flat surface for the tip/sample contact area.[13] The stiffness which corresponds to the slope of the initial unloading curve (See Figure S.7) is related to the Young modulus following Equation (1) with E_r, the reduced Young modulus, and A the contact area.[14,15].

$$S = \frac{dF}{dh} = \frac{2}{\sqrt{\pi}} E_r \sqrt{A}, \frac{1}{E_r} = \frac{(1-{\nu_t}^2)}{E_t} + \frac{(1-{\nu_s}^2)}{E_s} \tag{1}$$

where E_t, E_s and ν_t, ν_s are the Young modulus and Poasson ratio of the tip and sample, respectively. In our calculations, E_t = 300 GPa, ν_t = 0.3, and ν_s = 0.4. For a conical indenter in contact with a flat surface, the contact area is given by $A = \pi a^2 = \frac{4}{\pi} \tan(\theta)^2 h^2$ with $a = h_c \tan(\theta) = \frac{2}{\pi} \tan(\theta)$. h_c is the contact height and h the tip indentation. The force F is then related to h according to:

$$F = \frac{2E_r}{\pi} \tan\theta h^2 \tag{2}$$

Figure S.8 shows force measurements on untreated raw (UR) populus, where the loading force (red) during approach and unloading force (blue) during retraction are plotted against the tip indentation in the sample. In Figure S.8b, the red (blue) line represents the approach (retract) curve, displaying the adhesion-induced hysteresis. Noting pure elastic behavior is observed when loading (red) and unloading (blue) curves are dentical, in contrast, the force measurements in Figure S.8 contains both elastic and plastic deformation contributions. The elastic contribution is obtained from a linear fit of the beginning of the unloading curves.

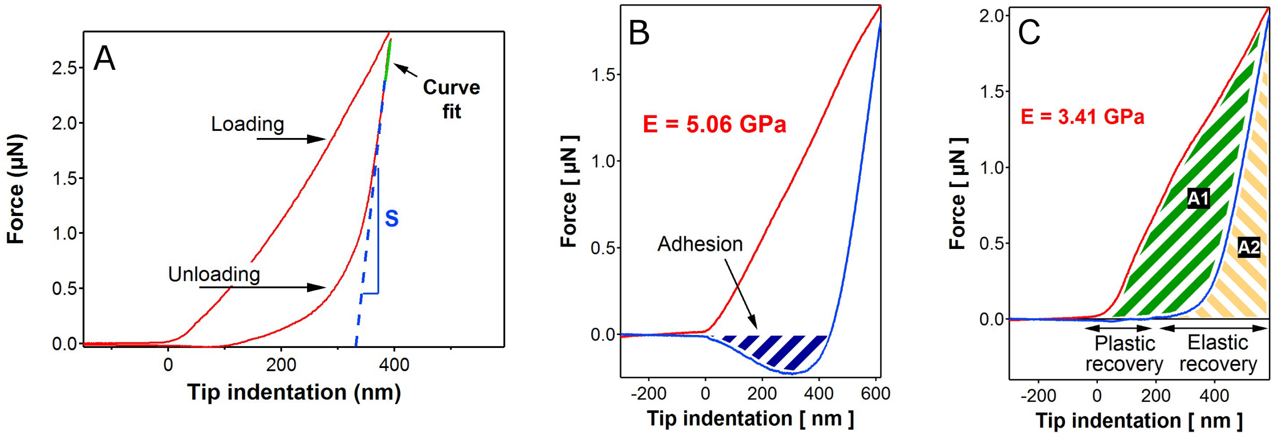

Figure S.8. Force measurements with AFM. Force-indentation curves describing experimental determination of adhesion, plastic, and elastic properties of the examined regions. **a**The young modulus is extracted numerically from fitting the beginning of the unloading curve with Equation (1). **b** The adhesion (depending on the probe-sample contact area and interfacial energies including that of the ambient tip-sample water meniscus) over a region with Young modulus of 5.06 GPa is numerically extracted from the lowest point (adhesion force) and the dashed area (adhesion energy) below the 0-force line in the indentation curve. **c** Another measurement over a region with Young modulus of 3.41 GPa, showing the plastic and elastic recovery with their respective proportions labeled A1 and A2.

References

1. Saito, K., Kato, T., Tsuji, Y. & Fukushima, K. Identifying the characteristic secondary ions of lignin polymer using tof-sims. *Biomacromolecules* **6**, 678–683 (2005).
2. Saito, K. *et al.* A new analysis of the depolymerized fragments of lignin polymer in the plant cell walls using tof-sims. *Applied Surface Science* **252**, 6734–6737 (2006).
3. Jung, S., Foston, M., Kalluri, U. C., Tuskan, G. A. & Ragauskas, A. J. Surface characterization of dilute acid pretreated populus deltoides by tof-sims. *Angew. Chem. Int. Ed.* **51**, 12005–12008 (2012).
4. Taylor, M. *et al.* 3d chemical characterization of frozen hydrated hydrogels using tof-sims with argon cluster sputter depth profiling. *Biointerphases* **11**, 02A301 (2016).
5. Aoyagi, S. Review of tof-sims bioanlysis using mutal information. *Surface and Interface Analysis* **41**, 136–142 (2009).
6. Henss, A., Hild, A., Rohnke, M., Wenisch, S. & Janek, J. *Biointerphases* **11**, 02A302 (2016).
7. Robinson, M. A. *et al.* Lipid analysis of eight human breast cancer cell lines with tof-sims. *Biointerphases* **11**, 02A303 (2016).
8. Jung, S., Foston, M., Sullards, M. C. & Ragauskas, A. J. Surface characterization of dilute acid pretreated populus deltoides by tof-sims. *Energy & Fuels* **24**, 1347–1357 (2010).
9. Zhou, C., Li, Q., Chiang, V. L., Lucia, L. A. & Griffis, D. P. Chemical and spatial differentiation of syringyl and guaiacyl lignins in poplar wood via time-of-flight secondary ion mass spectrometry. *Analytical Chemistry* **83**, 7020–7026 (2011).
10. Fletcher, J. S. & Vickerman, J. C. Secondary ion mass spectrometry: Characterizing complex samples in two and three dimensions. *Analytical Chemistry* **83**, 610–639 (2013).
11. Gindl, W. & Gupta, H. Cell-wall hardness and Young's modulus of melamine-modified spruce wood by nano-indentation. *Composites part A-Applied science and manufacturing* **33**, 1141–1145 (2002).
12. Zickler, G., Schoberl, T. & Paris, O. Mechanical properties of pyrolysed wood: a nanoindentation study. *Philosophical magazine* **86**, 1373–1386 (2006).
13. Sneddon, I. The relation between load and penetration in the axisymmetric Boussinesq problem for a punch of arbitrary profile.. *J. Eng. Sci.* **3**, 47–57 (1965).
14. Pharr, G.M., Oliver, W.C. & Brotzen, F.R. On the generality of the relationship among contact stiffness, contact area, and elastic modulus during indentation.. *J. Materials Research* **7**, 613–617 (1992).
15. Bulychev, S.I. & Alekhin, Z. Method of Kinetic Hardness and Microhardness in Testing Impression by an Indentor. *Lab* **53**, 76 (1987).
